# Muscular Localization of a Clear-Cell Renal Carcinoma: An Atypical Clinical Presentation

**DOI:** 10.7759/cureus.100314

**Published:** 2025-12-29

**Authors:** Nada Ebbadi, Mohamed Kaakoua, Hamza Laabbar, Mohamed Amine Haouane, Salah Ben Elhend, Moussa Abdoul Aziz Sawadogo, Soukayna Boujmadi, Abdelilah Mouhsine, Mohamed Amine Azami, Ismail Essadi

**Affiliations:** 1 Department of Radiology, Ibn Sina Military Hospital, Marrakech, MAR; 2 Department of Medical Oncology, Ibn Sina Military Hospital, Marrakech, MAR; 3 Department of Pathology, Cadi Ayyad University/Ibn Sina Military Hospital, Marrakech, MAR; 4 Department of Medical Oncology, Avicenne Military Hospital, Marrakech, MAR

**Keywords:** case report, clear-cell renal carcinoma, metastasis, secondary muscular metastases, unusual metastasis

## Abstract

Secondary muscular metastases of clear-cell renal carcinoma (CCRC) are very rare and exceptional. We report the case of a 67-year-old patient who presented clinically with back pain, a chronic cough, and a mass on the left arm. A thoraco-abdomino-pelvic CT scan showed the presence of a malignant renal process associated with multiple pulmonary lesions suggestive of metastases. The lung biopsy returned in favor of CCRC. Histological examination of the right arm mass confirmed the secondary muscular involvement of the renal process. The rarity of muscle metastases from CCRC represents a true diagnostic challenge, warranting systematic investigation.

## Introduction

Clear-cell renal carcinoma (CCRC) is the most common histological type of malignant kidney tumors (over 80%) [1]. The presence of synchronous metastases at the time of diagnosis occurs in 30% of cases [2]. The most commonly observed metastatic sites of CCRC are the lungs, bones, lymph nodes, liver, and adrenal glands [3]. Muscle metastases of CCRC are exceptional (less than 0.4%) [4,5]. The presence of muscle metastases in the arm is rarely reported in the literature. They generally present as a swelling of the arm, and the main differential diagnosis remains a second synchronous cancer. Diagnostic confirmation of secondary CCRC localization formally requires histological study with immunohistochemical analysis. Through the case of a patient with clear cell carcinoma with pulmonary and arm muscle metastases, we illustrate the diagnostic challenges associated with this unusual localization.

## Case presentation

**Figure 1:**
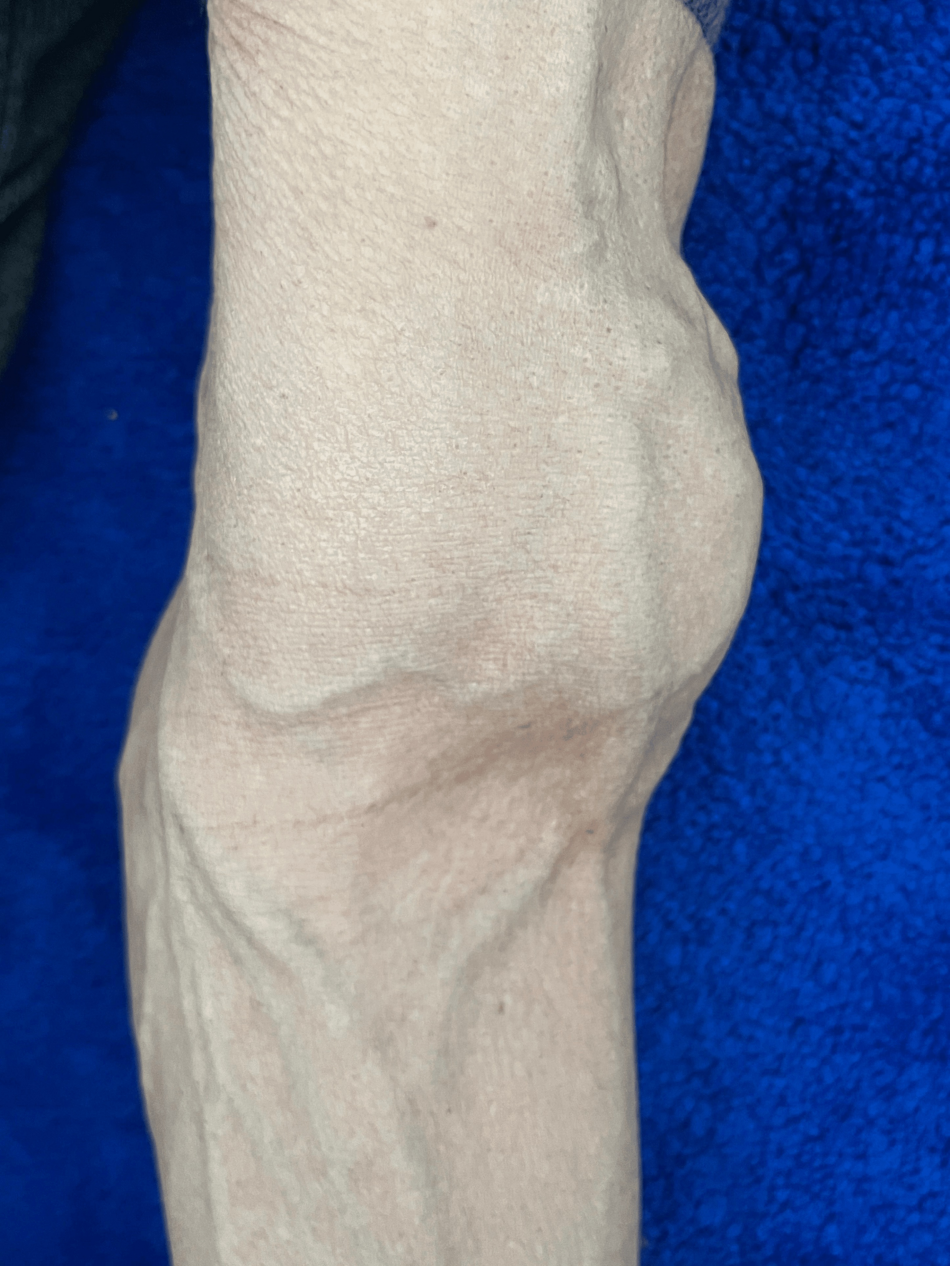
Swelling at the lower end of the left arm.

A 67-year-old patient, a chronic smoker of 20 pack-years with no other medical history, consulted for back pain and a chronic cough. There was no history of lumbar pain or hematuria. Clinical examination revealed a patient with a performance status of 1, digital clubbing, and the presence of a swelling at the lower end of the left arm without any inflammatory signs (Figure 1).

**Figure 2:**
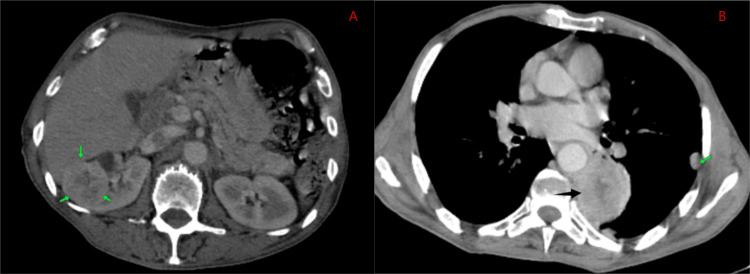
Thoraco-abdomino-pelvic CT scan. (A) Presence of a highly suspicious right renal mass (green arrow). (B) A mass in the dorsal segment of the culmen (black arrow), associated with a subpleural nodule (green arrow).

A thoraco-abdomino-pelvic CT scan showed the presence of a highly suspicious right renal mass measuring 42 × 40 mm, along with a mass in the dorsal segment of the culmen measuring 49 × 61 mm, associated with bilateral subpleural nodules (Figure 2). A CT-guided biopsy of the lung mass confirmed the diagnosis of a secondary localization of a CCRC.

**Figure 3:**
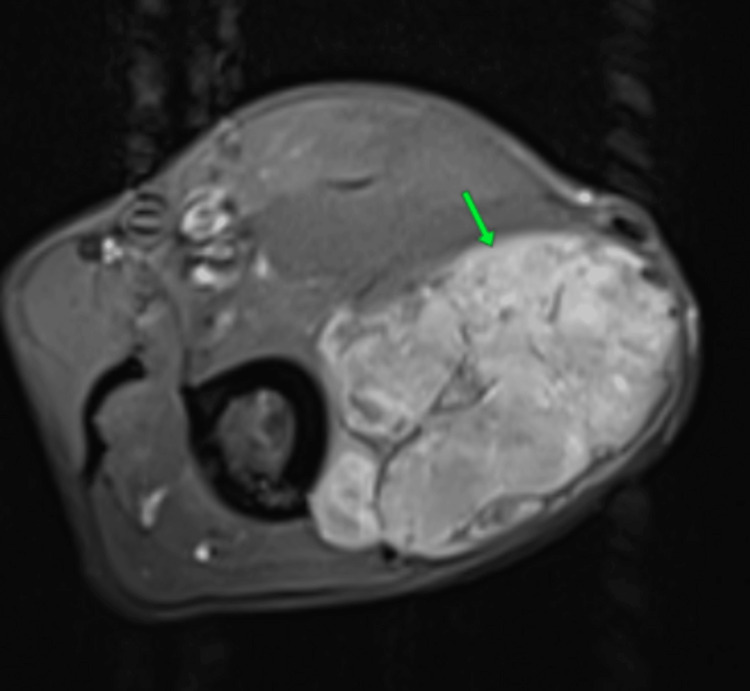
Axial MRI of the arm showing the presence of a mass involving the lateral head of the biceps muscle, hyperintense on T1 with homogeneous enhancement after gadolinium injection (green arrow).

Radiological examination of the swelling in the left arm by MRI revealed the presence of a mass involving the lateral head of the biceps muscle, showing high signal on T1 and T2 and homogeneously enhanced after gadolinium injection, measuring 63 × 43 mm (Figure 3).

**Figure 4:**
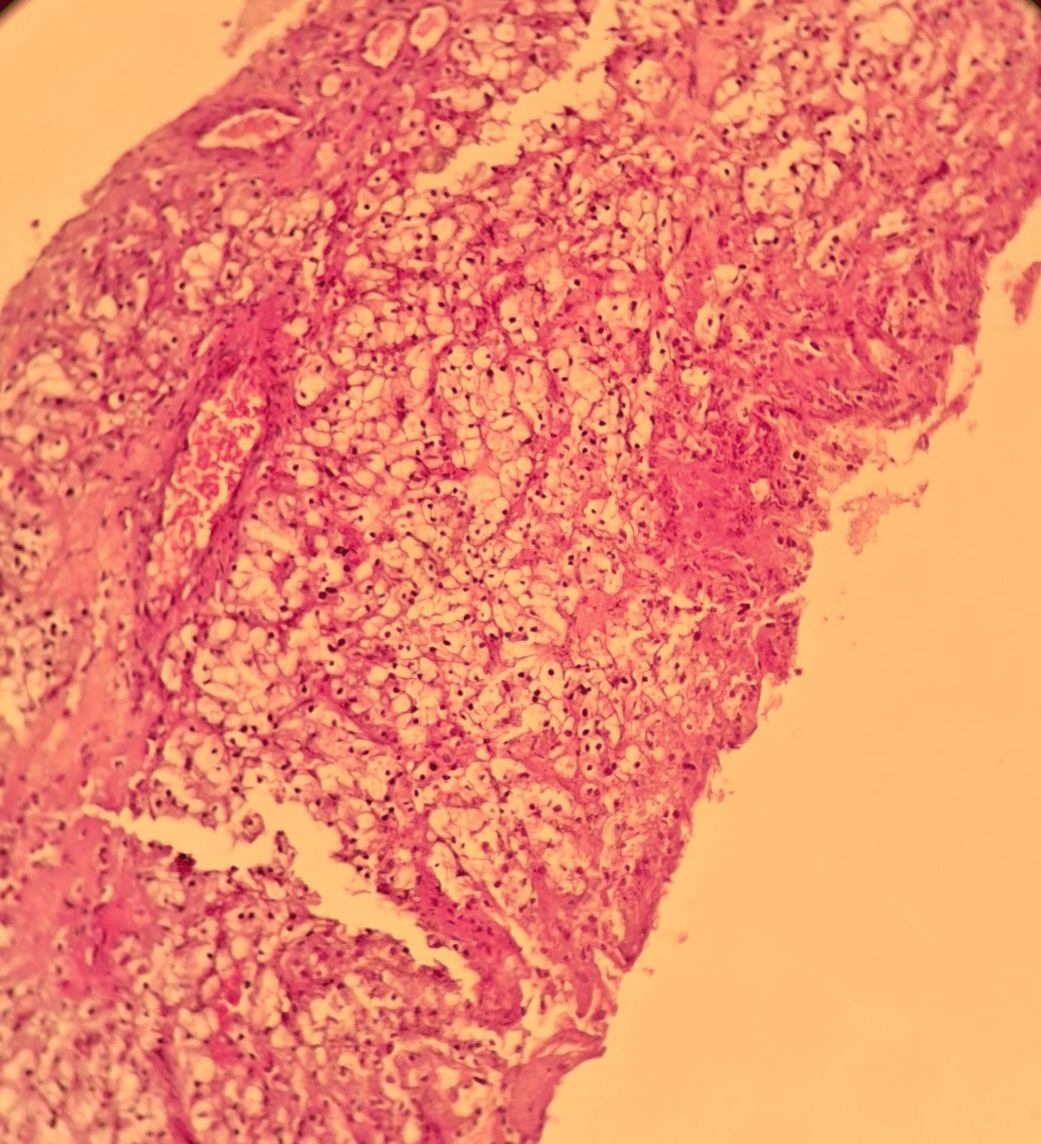
Microscopic image of a malignant tumor proliferation made up of nests and clusters of clear tumor cells (hematoxylin-eosin staining (×100).

**Figure 5:**
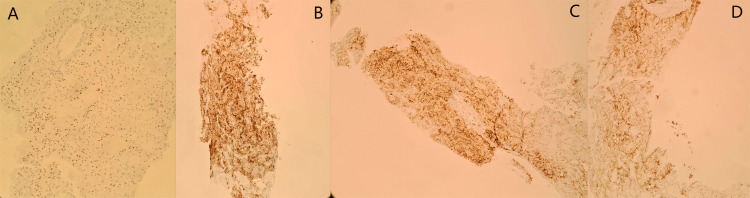
Immunohistochemical findings. (A) Expression of the anti-PAX8 antibody. (B) Expression of anti-pancytokeratin antibodies (AE1/AE3). (C) Expression of anti-RCC antibodies. (D) Expression of anti-CD10 antibodies.

The decision of the multidisciplinary consultation meeting was to perform a biopsy of the mass. The pathological study of the biopsied mass revealed a malignant carcinomatous tumor proliferation arranged in nests, clusters, and cords of medium-sized tumor cells with a round, mildly atypical nucleus and abundant clear cytoplasm (Figure 4). The immunohistochemical analysis performed showed positivity of the tumor cells for the following antibodies: pancytokeratin (AE1/AE3), PAX8, CD10, and RCC (Figure 5). Given this morphological appearance on hematoxylin and eosin staining and the immunohistochemical profile, the pathological study concluded a muscular localization of a CCRC, pending clinical and radiological correlation.

At the end of this investigation, the diagnosis of metastatic CCRC with pulmonary and muscular involvement was made. Prognostically, the patient had no poor prognostic criteria, notably no cytopenia on the blood count (hemoglobin at 126 g/L, platelets at 200 G/L, and neutrophils at 2,800 cells/µL) and no hypercalcemia (corrected blood calcium at 2.30 mmol/L). The disease was classified as good prognosis according to the HENG prognostic criteria. The patient was started on sunitinib at a dose of 50 mg per day for four weeks, followed by a two-week treatment-free period. Clinical and radiological evaluation after three treatment cycles showed stability of the target lesions.

## Discussion

Kidney cancer is the 14th most common cancer worldwide. It accounts for 3% of all cancers and is the third most common cancer of the urinary system [6]. More than one-third of patients are diagnosed at an advanced stage [2]. The most frequent secondary sites include the lungs, lymph nodes, bones, liver, adrenal glands, and brain. However, some unusual locations have been reported in the literature, notably the digestive tract, the genital system, head and neck, skin, and others [7]. The presence of muscular metastases from CCRC is estimated at less than 0.4% [4]. Hematogenous and lymphatic routes are the two main pathways for the spread of kidney cancer [8]. The mechanism behind the development of unusual secondary locations, particularly muscular ones, can be explained by the phenomenon of metastatic cascade [9]. The first metastatic site develops in the lungs, and from this site, a new location emerges [10,11]. Muscular metastases of CCRC can be either synchronous or metachronous [12].

The clinical presentation of this rare metastasis of CCRC varies depending on the muscle location. It often presents as a palpable muscle mass, muscle pain, ulceration, or even an incidental finding on radiological staging [13]. CT scanning remains insufficient to better characterize these secondary lesions. Muscle abscess and intramuscular hematoma are the main differential diagnoses [14]. MRI remains the gold standard for assessing the muscle lesion and its extent [14]. PET-CT remains more sensitive for mapping muscle lesions [15]. Only histological examination can confirm the diagnosis of a secondary muscle localization of CCRC.

Currently, the treatment of CCRC with muscle metastases remains primarily systemic therapy based on an anti-angiogenic and immunotherapy combination (lenvatinib-pembrolizumab or axitinib-pembrolizumab or cabozantinib-nivolumab) or dual immunotherapy (ipilimumab-nivolumab). For patients who are not eligible for immunotherapy and are at low risk, anti-angiogenic therapy (sunitinib or pazopanib) remains a therapeutic option [16]. The prognosis of this disease remains poor, although it has improved with new therapies, with an overall survival reaching just over 40 months [16].

## Conclusions

Muscle metastases of CCRC remain a very rare secondary location, which poses a real diagnostic challenge. Several differential diagnoses can be considered, particularly a second cancer, especially soft-tissue sarcomas. Only histological confirmation can establish the diagnosis. A histological study with an immunohistochemical complement must be performed whenever possible. The management of these muscle metastases is non-specific and follows the treatment of systemic disease.

## References

[1] Escudier B, Porta C, Schmidinger M (2019). Renal cell carcinoma: ESMO Clinical Practice Guidelines for diagnosis, treatment and follow-up†. Ann Oncol.

[2] Dabestani S, Thorstenson A, Lindblad P, Harmenberg U, Ljungberg B, Lundstam S (2016). Renal cell carcinoma recurrences and metastases in primary non-metastatic patients: a population-based study. World J Urol.

[3] Dudani S, de Velasco G, Wells JC (2021). Evaluation of clear cell, papillary, and chromophobe renal cell carcinoma metastasis sites and association with survival. JAMA Netw Open.

[4] Singla A, Sharma U, Makkar A (2022). Rare metastatic sites of renal cell carcinoma: a case series. Pan Afr Med J.

[5] Safadi A, Abu Ahmad MS, Sror S, Schwalb S, Katz R (2018). Simultaneous metachronous renal cell carcinoma and skeletal muscle metastasis after radical nephrectomy. Urol Case Rep.

[6] Bigot P, Boissier R, Khene ZE (2024). French AFU Cancer Committee Guidelines - update 2024-2026: management of kidney cancer. Prog Urol.

[7] Vidart A, Fehri K, Pfister C (2006). [Unusual metastasis of renal carcinoma]. Ann Urol (Paris).

[8] Pagano S, Franzoso F, Ruggeri P (1996). Renal cell carcinoma metastases. Review of unusual clinical metastases, metastatic modes and patterns and comparison between clinical and autopsy metastatic series. Scand J Urol Nephrol.

[9] Akhtar M, Haider A, Rashid S, Al-Nabet AD (2019). Paget's "Seed and Soil" theory of cancer metastasis: an idea whose time has come. Adv Anat Pathol.

[10] Maja S, Danica N, Aleksandra B (2024). [Uncommon muscle metastatic sites of renal cell carcinoma]. Srpski Arhiv za Celokupno Lekarstvo.

[11] Chowdhury S, Haque S, Sanekommu H, Nightingale B, Razi S, Hossain MA (2024). An atypical presentation of metastatic renal cell carcinoma. J Med Cases.

[12] Murugan N, Figueroa Hernandez Y, Amin N, Dahip M, Daglilar E, Chela HK (2025). Uncommon metastatic pattern of renal cell carcinoma (simultaneous metastasis to the small intestine and skeletal muscle): a case report. World J Gastrointest Pharmacol Ther.

[13] Amro SY, Kassas M, Hijazi M, Natout M (2025). Late recurrence of renal cell carcinoma presenting with skeletal muscle metastases: a case report. Cureus.

[14] Haygood TM, Sayyouh M, Wong J, Lin JC, Matamoros A, Sandler C, Madewell JE (2015). Skeletal muscle metastasis from renal cell carcinoma: 21 cases and review of the literature. Sultan Qaboos Univ Med J.

[15] Ben Nasr M, Ben Hamida O, Somai M, Yeddes I, Slim I, Meddeb I, Mhiri A (2023). [Interest of 18F-FDG PET-CT in detecting atypical muscle metastases in bronchopulmonary cancer: about 5 cases]. Méd Nucl.

[16] Powles T, Albiges L, Bex A (2024). Renal cell carcinoma: ESMO Clinical Practice Guideline for diagnosis, treatment and follow-up. Ann Oncol.

